# Protocol: Adaptive Implementation of Effective Programs Trial (ADEPT): cluster randomized SMART trial comparing a standard versus enhanced implementation strategy to improve outcomes of a mood disorders program

**DOI:** 10.1186/s13012-014-0132-x

**Published:** 2014-09-30

**Authors:** Amy M Kilbourne, Daniel Almirall, Daniel Eisenberg, Jeanette Waxmonsky, David E Goodrich, John C Fortney, JoAnn E Kirchner, Leif I Solberg, Deborah Main, Mark S Bauer, Julia Kyle, Susan A Murphy, Kristina M Nord, Marshall R Thomas

**Affiliations:** VA Center for Clinical Management Research, VA Ann Arbor Healthcare System, 2215 Fuller Rd, Mailstop 152, Ann Arbor, 48105 MI USA; Department of Psychiatry, North Campus Research Complex, University of Michigan Medical School, 2800 Plymouth Rd, Bldg 16, Ann Arbor, 48109-2800 MI USA; Institute for Social Research, University of Michigan, 426 Thompson Street, Ann Arbor, 48104-2321 MI USA; Department of Health Management and Policy, School of Public Health, University of Michigan, 1415 Washington Heights, Ann Arbor, 48109-2029 MI USA; Colorado Access, 10065 E. Harvard Ave, Suite 600, Denver, 80231 CO USA; Department of Psychiatry, University of Colorado School of Medicine, 13199 East Montview Blvd, Mailstop F550, Suite 330, Aurora, 80045 CO USA; Seattle HSR&D Center of Innovation for Veteran-Centered and Value-Driven Care, VA Puget Sound Health Care System, 1660 S. Columbian Way, S-152, Seattle, 98108 WA USA; VA Mental Health Quality Enhancement Research Initiative (MH QUERI), North Little Rock, 27114 AR USA; Department of Psychiatry, College of Medicine, University of Arkansas for Medical Sciences, 4301 W. Markham, Little Rock, 72205 AR USA; HealthPartners Institute for Education and Research, 3311 E. Old Shakopee Road, Bloomington, 55425 MN USA; Department of Health and Behavioral Sciences, University of Colorado Denver, Denver, 80217 CO USA; VA Center for Healthcare Organization and Implementation Research, VA Boston Healthcare System, Bldg 9, Jamaica Plain Campus, 150 South Huntington Ave (152 M), Boston, 02130 MA USA

**Keywords:** Adaptive intervention, Depression, Health behavior change, Care management

## Abstract

**Background:**

Despite the availability of psychosocial evidence-based practices (EBPs), treatment and outcomes for persons with mental disorders remain suboptimal. Replicating Effective Programs (REP), an effective implementation strategy, still resulted in less than half of sites using an EBP. The primary aim of this cluster randomized trial is to determine, among sites not initially responding to REP, the effect of adaptive implementation strategies that begin with an External Facilitator (EF) or with an External Facilitator plus an Internal Facilitator (IF) on improved EBP use and patient outcomes in 12 months.

**Methods/Design:**

This study employs a sequential multiple assignment randomized trial (SMART) design to build an adaptive implementation strategy. The EBP to be implemented is life goals (LG) for patients with mood disorders across 80 community-based outpatient clinics (*N* N = 1,600 patients) from different U.S. regions. Sites not initially responding to REP (defined as <50% patients receiving ≥3 EBP sessions) will be randomized to receive additional support from an EF or both EF/IF. Additionally, sites randomized to EF and still not responsive will be randomized to continue with EF alone or to receive EF/IF. The EF provides technical expertise in adapting LG in routine practice, whereas the on-site IF has direct reporting relationships to site leadership to support LG use in routine practice. The primary outcome is mental health-related quality of life; secondary outcomes include receipt of LG sessions, mood symptoms, implementation costs, and organizational change.

**Discussion:**

This study design will determine whether an off-site EF alone versus the addition of an on-site IF improves EBP uptake and patient outcomes among sites that do not respond initially to REP. It will also examine the value of delaying the provision of EF/IF for sites that continue to not respond despite EF.

**Trial registration:**

ClinicalTrials.gov identifier: NCT02151331

**Electronic supplementary material:**

The online version of this article (doi:10.1186/s13012-014-0132-x) contains supplementary material, which is available to authorized users.

## Background

It can take years to translate evidence-based practices (EBPs) into community-based settings [1]. This research-to-practice gap is especially pronounced for psychosocial EBPs for mood disorders (depression, bipolar disorders), which represent the top ten causes of disability according to the World Health Organization. Mood disorders are associated with significant functional impairment, high medical costs, and preventable mortality [2],[3]. For many patients, pharmacotherapy is not enough to improve outcomes, with psychosocial treatments in addition to pharmacotherapy recommended [4]-[7].

Despite the availability of psychosocial EBPs [1],[4]-[8] for mood disorders, they rarely get implemented and sustained in community-based practices [9]-[11]. This is primarily due to a lack of available strategies to help providers embed the EBP into routine clinical workflows, and garner support from clinical and administrative leadership on the EBP’s added value to the practice [8],[12]-[25]. As a result, outcomes for persons with mental disorders remain suboptimal [12],[26]. New healthcare initiatives including medical home models designed to improve efficiency and value have not included specific strategies to assist local providers in implementing EBPs in routine care [27]-[29]. Moreover, up to 98% of patients with mood disorders receive care from smaller clinical practices, which may not have the tools to fully implement medical homes [30].

**Figure 1:**
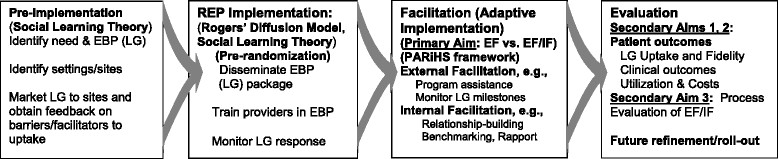
**Replicating Effective Programs (REP) and Internal/External Facilitation (EF/IF).**

For EBPs to reach these patients, effective implementation strategies are needed [31]-[34]. Implementation strategies that have been highly specified, operationalized, and previously used to implement EBPs into usual care settings include Replicating Effective Programs (REP) [35]. REP primarily focuses on standardization of the EBP implementation into routine care settings through toolkit development and marketing, provider training, and program assistance (Figure 1). Used successfully to improve the uptake of brief HIV interventions [36]-[39], REP is a low-intensity intervention with minimal costs for sites. However, when applied to the implementation of a psychosocial EBP for mood disorders, REP resulted in less than half of sites implementing the EBP [40].

Recognizing the need for more intensive implementation strategies that leverage local provider initiative and outside expertise [41], REP was enhanced to include Facilitation [40],[41]. Two Facilitation roles evolved: an External Facilitator (EF), employed from outside the local site, and an Internal Facilitator (IF), employed at each of the local sites. EFs are situated at a central location and provide technical expertise and program support in implementing the EBP at the local site. In contrast, IFs have a direct reporting relationship to site leadership and have protected time to support providers in implementing EBPs by helping them align the EBP activities with the priorities of the clinic providers and local leadership.

A recent randomized controlled trial [40],[42] of added an External Facilitator (REP + EF) versus REP alone found that among 88 sites not responding to REP, sites randomized to receive REP + EF compared to sites continuing with REP were more likely to adopt the EBP within 6 months (defined as percentage of patients receiving care management: 56% vs. 28%) [42]. This suggests that while REP + EF led to increased uptake, a more intensive, locally oriented implementation strategy (e.g., Internal Facilitation) might be needed for EBP uptake in sites that do not fully implement the EBP. In a different study, REP in combination with EF and IF (REP + EF/IF) versus standard REP alone improved adoption and fidelity to a mood disorder EBP [43],[44], underscoring the promise of REP + EF/IF.

However, because IF requires additional time commitment from sites, and not all sites may need IF, an *adaptive implementation strategy* approach is a more practical design. In contrast to measuring implementation non-response (or correlates of it) and not using it to guide improved implementation and outcomes, in adaptive implementation strategies, implementation interventions are augmented in direct response to limited adoption of EBPs among specific sites based on circumstances that may not be observable at baseline. This study addresses key scientific questions that need to be answered in order to develop an effective adaptive implementation strategy. Notably, among sites not responding to REP (i.e., not adopting the EBP), is it best to augment with REP + EF/IF or with REP + EF, or is it best to delay the provision of REP + EF/IF for sites that continue not to respond despite an initial REP + EF augmentation?

### Aims and objectives

The overarching goal of this study is to build the most effective adaptive implementation strategy involving two cutting-edge implementation strategies (REP and Facilitation) to improve practice-level uptake of EBPs and patient outcomes. The EBP, life goals (LG), is an evidence-based psychosocial treatment delivered in six individual or group sessions that was shown in seven randomized controlled trials to improve mental and physical health outcomes among patients with mood disorders [44]-[49]. We will use a sequential multiple assignment randomized trial (SMART) [50],[51] design to build the adaptive intervention in which data on early response or non-response to REP will be used to determine the next implementation strategy.

#### Primary study aim

The primary aim of this study is to determine in a SMART implementation trial, among patients with mood disorders in sites that do not exhibit response to REP alone after 6 months (i.e., <50% patients receive ≥3 LG sessions), the effect of adding an External and Internal Facilitator (REP + EF/IF) versus REP + EF on patient-level changes in mental health-related quality of life (MH-QOL; primary outcome), receipt of LG sessions, mood symptoms, implementation costs and organizational change (secondary outcomes) from month 6 to month 24.

#### Secondary aim 1

The first secondary aim is to determine, among REP + EF sites that continue to exhibit non-response after an additional 6 months, the effect of continuing REP + EF vs. REP + EF/IF on patient-level changes in the primary and secondary outcomes from month 12 to month 24.

#### Secondary aim 2

The second secondary aim is to estimate the site-level costs of REP + EF/IF compared to REP + EF.

#### Secondary aim 3

The third secondary aim is to describe the implementation of EF and EF/IF at the site level, including interaction between the two roles and the specific strategies EFs and IFs use to facilitate LG uptake across different sites.

The result of this current SMART study will be an optimized, adaptive implementation strategy that that could be applied to healthcare systems to improve outcomes for patients with mental disorders.

## Methods

**Figure 2:**
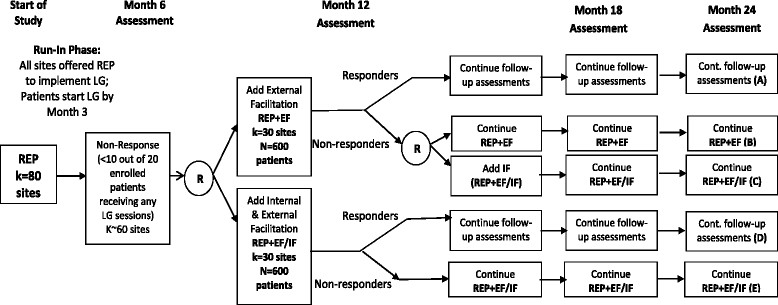
**SMART trial design of REP combined with External (EF, REP + EF) and Internal Facilitation (IF, REP + EF/IF).**

This cluster randomized SMART implementation trial (Figure 2) involves community-based clinics (sites) from Michigan (MI) and Colorado (CO) that care for persons with mood disorders (depression or bipolar disorders). This study was reviewed and approved the local institutional review boards.

### Setting

Eighty community-based mental health or primary care clinics from the two states will be recruited to participate in the study based on lists of primary care and community mental health programs available from state organizations. Since the EBP to be implemented (LG) has been shown to be effective in improving outcomes across different settings (primary care, community mental health) [45]-[47],[49],[52], a diverse array of sites will be recruited to maximize study generalizability.

### Sites

Site inclusion criteria include the following:

1. Community-based mental health or primary care clinic located in Michigan or Colorado with at least 100 unique patients diagnosed with or treated for mood disorders.
2. Availability of a bachelor’s- or master’s-level healthcare provider with a mental health background and experience with implementing individual or group sessions (core modality of LG) who can be trained to provide LG to up to 20 adult patients with mood disorders in the clinic in a 1-year period.
3. Availability of an employee at the site with direct reporting authority to the leadership of the site or parent practice organization who could serve as potential internal facilitator.

### Study design flow

Primary care or mental health outpatient clinics confirming eligibility using a standard organizational assessment and agreeing to participate will initially be offered REP for 6 months in order to implement LG (Figure 2). Based on previous research [53] and preliminary data, it is expected that after 6 months of REP, at least 80% of sites will be non-responsive to REP. The primary focus of the randomized comparisons in this study are sites that are initially non-responsive to REP, defined based on our preliminary studies as <50% of previously identified patients receiving at least three LG sessions (≥3 out of 6) to achieve minimum clinically significant results (85-89). Although not part of the primary randomized comparisons, sites that are responsive at 6 months will continue to be followed and outcomes will be assessed.

### Patients within sites

The unit of analysis is LG eligible patients within sites who are diagnosed with or treated for mood disorders. Patients are identified during the first 6 months as part of REP, i.e., prior to site-level randomization (see step 1 of REP below). Patient-level primary and secondary outcomes will be assessed by independent evaluators (i.e., study associates who are not aware of the assignment to REP + EF or REP + EF/IF) across all sites prior to identifying site response/non-response status at month 6, prior to identifying site response/non-response status at month 12, and at months 18 and 24.

### Site randomization

The unit of intervention (randomization) is the site. Sites that are not responding to REP at month 6 will be randomized 1:1 by the study data analyst to receive additional External Facilitation (REP + EF) or External plus Internal Facilitation (REP + EF/IF). After another 6 months (at month 12), (i) REP + EF sites that are still non-responsive (defined as <50% patients receiving ≥3 life goals sessions) will be randomized 1:1 to either continue REP + EF or augmentation with IF (REP + EF/IF) for an additional 12 months, and (ii) intervention will be discontinued for sites that are responsive. The month 6 randomization will be stratified by state, practice type (primary care or mental health site) [54],[55], and by site-average baseline MH-QOL (e.g., low (<40) vs. high (≥40)) at baseline. The month 12 randomization will be stratified by state, practice type, and site-average MH-QOL at month 12. This will ensure that intervention groups are balanced for site variables that may correlate highly with outcomes. The study analyst will generate the stratified permuted-block random allocation lists (blocks of size 2, 4, and 6) using a computer program such as PROC PLAN in SAS. Sites are expected to become eligible and accrue in groups of 6-12 (staggered entry). A site is considered randomized once the study analyst informs the study coordinator of each site’s random assignment.

### Evidence-based practice to be implemented

LG [44]-[49],[52] is a psychosocial intervention for mood disorders delivered in six individual or group sessions (Table 1). Based on social cognitive theory [56]-[58], LG encourages active discussions focused on individuals’ personal goals that are aligned with healthy behavior change and symptom management strategies. LG was chosen to be the EBP to implement because mood disorders are common in both community-based primary care and mental health clinics [59],[60], are considered to be high-priority populations based on input from community partners, and because LG was shown to improve outcomes including mental health-related quality of life in this group [45]-[47],[49],[52]. Compared to usual care, LG improved outcomes among a cross-diagnosis sample of community-based outpatients with mood disorders [45]-[49],[52], notably a four-point increase in mental and physical health-related quality of life scores based on the SF-12 (e.g., Cohen’s *D* = .36) [45],[46],[49]. LG has been shown to be equally effective in patients with co-occurring substance use and medical comorbidities [46],[47],[49],[52],[61]. Community-based providers helped to adapt LG [46]-[48], but as with many psychosocial EBPs, have not been widely implemented in smaller practices [62].

**Table 1:** **Components of the life goals program**

Component	Description/core concepts across all self-management sessions
Self-management sessions	A minimum of six individual sessions lasting ~50 min each or four group sessions lasting ~90 min each focused on active discussions around personal goals, mental health symptoms, stigma, and health behaviors
Values	Explore personal values and their relationship to health change behavior; also explore types of stigma and ways to overcome stigma
Collaborative care	Identify ways to strengthen relationships and participation in healthcare, explore the importance of coming prepared to medical visits, and identify ways to ensure true collaboration is occurring to support optimal health outcomes.
Self-monitoring	Discuss the importance of measurement-based care and develop a meaningful self-monitoring plan for identified mental or physical health condition (i.e., PHQ-9, GAD-7, sleep log, food tracking)
Symptom profile	Create a personal symptom profile for a specific mental health condition with a focus on identifying early warning signs and ways to use this knowledge to bolster self-management skills
Triggers	Identify positive and negative, preventable and inevitable triggers and ways to better prepare for and manage these triggers
Cost/benefit analysis of responses	Explore previous responses to psychiatric symptoms and link responses to personal values and behavior change goals
Life goals	Create SMART goals, problem-solve barriers, and ensure personal goals are in line with identified values
Care management	At least monthly (for 6 months) individualized follow-up contacts to support lessons learned; contacts work to problem-solve barriers to health-behavior goals and provide additional support for mental health issues interfering with goal attainment
Provider decision support	Knowledge and availability of best practice treatments for mental and physical health conditions either in-house or via consult

### Implementation strategies

#### REP

All sites will receive REP (Table 2) in the first 6 months. REP is based on the Centers for Disease Control and Prevention’s (CDC’s) Research to Practice Framework project [35],[62],[63] and includes initial marketing of the LG program by study investigators, package dissemination and training by an off-site trainer, as-needed program support in using LG (program assistance), and LG uptake monitoring for up to 6 months [35],[44]. The theories underlying REP [64],[65] include Rogers’ Diffusion of Innovations [36] and Social Learning Theory [66]. For this study, REP includes the following components:

**Table 2:** **Description of REP, REP + EF, and REP + EF/IF implementation strategies to enhance the uptake of the life goals (LG) evidence-based practice**

Implementation component	REP	REP + EF	REP + EF/IF
REP (REP)	All sites	Randomize to non-responding sites	Randomize to non-responding sites
Step 1: Market LG, disseminate LG package. (a) Marketing (pre-implementation): hold pre-implementation meetings with site representatives to describe the benefits of LG and identify potential LG providers and internal facilitators (IF). (b) Dissemination: regional in-services to disseminate LG implementation guide to providers, schedule trainings.	**√**	**√**	**√**
Step 2: Train site providers in LG. Conduct regionalized 8-h training for LG providers covering LG session content and delivery via LG website as well as patient tracking and monitoring over time.	**√**	**√**	**√**
Step 3: As-needed program assistance and LG uptake monitoring via secure web-based reporting sheets. *Ad hoc* program support available by study program support assistant.	**√**	**√**	**√**
REP + External Facilitator (REP + EF)			
Step 1: Initiation and benchmarking: EF contacts each LG provider, works with LG provider to identify potential barriers and facilitators to LG uptake from the LG provider’s perspective, and sets measurable goals to uptake.		**√**	**√**
Step 2: Coaching: EF makes calls on a biweekly basis to site’s LG providers to develop rapport and provide specific guidance on overcoming barriers in implementing LG components by aligning LG’s strengths with LG’s available influence at the site. If needed, EF refers LG provider to study LG program support assistant.		**√**	**√**
Step 3: Public recognition of “bright spots” (high-performing sites): EF provides state-specific report on sites’ progress and disseminates LG provider success stories.		**√**	**√**
REP + External and Internal Facilitator (REP + EF/IF)			
Step 1: Initiation and benchmarking: EF contacts each LG provider and holds call with LG provider and IF to give background on LG, review potential barriers and facilitators to LG uptake, and set measurable goals to LG uptake.			**√**
Step 2: Leveraging: IF meets with LG provider biweekly, identifies local site priorities per leadership input, identifies other LG program champions, and helps LG provider summarize and describe added value of LG to leadership and site providers (e.g., consistency with other initiatives, support from leadership).			**√**
Step 3: Coaching: IF, EF, and LG hold biweekly calls to develop rapport; EF provides guidance to LG on overcoming specific barriers to LG uptake by aligning LG’s strengths with LG’s available influence at the site (EF), and IF aligns goals of LG provider with existing site priorities, based on feedback from site leadership. If needed, EF refers LG provider to study LG program support assistant.			**√**
Step 4: Ongoing marketing: IF, leadership, and LG provider summarize progress and develop business and sustainability plans.			**√**

#### Step 1: marketing and dissemination of LG package, in-service, and patient selection

Regional in-services will first be provided by study investigators who will give an overview of LG including the evidence, and details on how to implement LG in their setting. Each site will designate at least one provider with a mental health background to implement LG (“LG provider”), and the LG package will be disseminated by the REP trainer to these providers.

The LG REP package includes all of the components needed to implement LG, including the LG provider manual; a protocol for identifying patients to enroll in LG; LG session scripts and focus points covered in each session in a semi-directed fashion; the registry template for tracking enrolled patients’ progress in LG session-based personal goals, symptoms, and health behavior change; scripts for follow-up calls, patient workbooks, and an implementation manual describing logistics (e.g., identifying rooms if group sessions are used, identifying patients for LG, medical record templates for LG sessions, billing codes). Because LG effectiveness was already demonstrated in patients with mood disorders and that the primary goal of the study is to promote LG implementation, LG providers will identify and offer LG to patients at their site with mood disorders based on guidance from the REP trainer [44],[62]. The trainer will work with LG providers to identify up to 30 patients within a 3-month period who are appropriate for the LG program using the following criteria that are included in the registry template:

1. adults 21 years or older with a diagnosis and current documentation of antidepressant or mood stabilizer for a mood disorder (depression or bipolar disorder) based on medical record review
2. not currently enrolled in residential treatment
3. can understand English and have no terminal illness or cognitive impairment that precludes participation in outpatient psychosocial treatment based on confirmation by the treating clinician.

Assuming a 33% refusal or ineligibility rate [47], as well as a potential 12-month attrition rate of around 20% from prior studies [47], LG providers will be expected to identify and initially offer LG to 30 patients, assuming 20 will initially participate, with 16 to complete 12-month follow-ups.

#### Step 2: REP LG training and provider competency

LG providers will undergo a 1-day training program that has been provided to over 200 clinicians nationally [44],[67]-[69]. The LG trainer will first provide an orientation to the evidence behind LG and core elements and then a step-by-step walk-through of LG components. The trainer will then demonstrate each LG session and follow-up contact procedures, as well as the standardized criteria for identifying patients who are appropriate for LG in routine practice. LG providers will break into groups and practice each component.

After identifying eligible patients, LG providers will make an initial contact to each patient, introduce the LG program, and schedule in-person group or individual sessions. Individual sessions typically last 50 min each, while group sessions typically last around 90 min to allow for additional discussions between group members. Participants can make up sessions over the phone if they are unable to make in-person sessions. In the sessions, the LG provider will encourage active discussions that progressively have participants identify a personal symptom profile and triggers to the mood symptom or episode and develop an activity plan for identifying warning signs of symptoms and an activity plan for adopting a specific health behavior to mitigate symptoms and promote wellness. Participants will be given a workbook with exercises on behavior change goals, symptom assessments, and coping strategies. The LG provider will also make individual contacts to patients after the end of the sessions on a regular basis to review symptoms and behavior change. LG providers are trained to handle patients with elevated symptoms or suicidal ideation as part of their clinical responsibilities.

#### Step 3: As-needed REP program assistance and LG monitoring

The final phase of REP consists of as-needed program assistance provided to LG clinicians who contact the LG program support specialist. In addition, each LG provider is sent a standard monitoring form on a biweekly basis for up to 6 months to have them record the number of patients approached and number receiving each LG session. Monitoring forms will be used to assess non-response across sites and will be corroborated based on patient self-reported LG use from the follow-up surveys (see data collection below). Sites will receive feedback reports from the program support assistant that includes performance on uptake measures (i.e., how many enrolled, how many sessions) in comparison to other sites. Also, sites will receive periodic (quarterly) newsletters about the study progress.

#### Facilitation

The Facilitation implementation strategy will consist of EF and IF (Table 2). Based on the Promoting Action on Research Implementation in Health Services (PARiHS) Framework [70]-[74], facilitation is defined as the process of interactive problem solving and support that occurs in the context of a recognized need for improvement and a supportive interpersonal relationship [41],[74].

LG providers from sites that are not responsive to REP in 6 months after initiating LG at their site will be randomized to receive REP + EF or REP + EF/IF. Sites receiving REP + EF will be contacted by the EF, who resides off site. The EF will contact individual providers from each site on a regular basis and conduct 1-hour phone calls to provide guidance on implementing LG components. Notably, the EF will be trained to identify individual strengths of each LG provider and help leverage those strengths with available influence the LG provider has at the site. The EF will also set measurable objectives in implementing LG (e.g., number of patients completing at least one group session), review implementation progress, and where appropriate, refer the provider to the study program support assistant for specific guidance on LG use (Table 2).

LG providers from sites that are not responsive to REP initially and randomized to receive REP + EF/IF will also be contacted by the EF, and the study team will also contact the site to identify an IF at the time of randomization. The IF will be an individual supervising the LG provider in some capacity. In this arm, LG providers will have regular calls with the EF and IF, as well as meet with IF one on one on a regular basis. As with the REP + EF arm, EFs will identify the individual strengths of each LG provider and help leverage those strengths with available influence the LG provider has at the site. In addition, the IF at each site will meet with the LG provider and will use their internal knowledge of the site to help the LG provider identify opportunities to align LG program goals with existing site priorities (Table 2). Sites randomized to receive IF will receive up to $5,500 to cover IF-related time.

### Ensuring fidelity to implementation strategies

Fidelity monitoring will be used to assess whether each site is receiving the core components of each implementation strategy (REP, EF, and IF) and to ensure that there is no contamination across roles. Data from LG provider logs and EF/IF activities completed by study staff will be used to ascertain fidelity within each 6-month period of implementation strategy exposure using established checklists. All sites will get the same LG REP package, and LG training will be conducted by the study trainer who will hold regional trainings in each state. The EF will be trained by study investigators based on a 2-day training program developed for national roll-out of both the REP and EF/IF programs.

Fidelity to REP is defined based on number of sites receiving an LG package, number of providers completing the 1-day LG training program, and number of completed lists of patients receiving LG. Fidelity to the EF role will be defined using the following criteria based on the components outlined in Table 2: 1) number of completed calls by the EF with LG providers at each site; 2) number of documented barriers, facilitators, and specific measurable goals to LG uptake; and 3) documentation of LG provider strengths and available opportunities to influence site activities and overcome barriers. Fidelity to the IF role is defined as 1) number of meetings with the LG provider and EF, 2) number of meetings IF and LG provider have with site leadership, 3) number of documented opportunities to leverage LG uptake with existing site priority goals, and 4) development of a strategic plan to implement LG by the IF.

### Primary outcomes and measures

Study staff members (not site employees) will collect patient and provider data to ensure consistency of outcomes data collection over time. Outcomes assessors will be blinded to implementation condition. The assessment package previously implemented by study investigators was informed by the RE-AIM framework for evaluating implementation of EBPs and includes measures used in routine clinical care [75],[76]. Key measures include patient-level outcomes, LG fidelity, organizational factors, and REP, EF, and IF activities [49],[77]-[79].

### Patient data collection

Lists of patients and contact information that were identified by the LG providers during pre-randomization will be sent to study staff members who will conduct independent clinical assessments. As the study is focused on implementation processes for an evidence-based treatment that does not involve randomizing at the patient level, local institutional review boards (IRBs) considered the protocol to not fall under research, and no patient informed consent is required for the clinical assessments. Staff members will contact patients and describe the purpose of the phone-based clinical assessment, which will be conducted at baseline, 6, 12, 18, and 24 months later.

The clinical assessment will include previously established measures used in routine care, including the SF-12 for MH-QOL [80] (primary outcome), the Patient Health Questionnaire (PHQ-9) for mood symptoms [81],[82], the World Health Organization Disability Assessment Scale (WHO-DAS) for functional impairment [83],[84], GAD-7 for anxiety symptoms [85], and the Internal State Scale for manic symptoms [86],[87] (secondary and exploratory outcomes). Additional information [44],[88]-[91] including participant demographics will also be ascertained from the clinical assessment during each follow-up period.

### Provider and organizational surveys

Study staff members will survey providers from the participating sites, including the site clinical director and LG providers on organizational factors that might impact implementation and outcomes. As the study’s principal institutional review board (University of Michigan Medical School Institutional Review Board - IRBMED) considered this study to fall under quality improvement activities, no informed consent is required from providers or clinic directors. Clinic directors will be contacted prior to the initiation of the REP in-service to complete an initial site organizational survey [43],[44] that includes questions on resources, staff turnover, and integrated care [92]. A longitudinal organizational assessment will also be given to the clinic directors and LG providers prior to the initiation of REP, then again at 12 and 24 months later to assess changes in organizational features that might be impacted by REP and Facilitation. The assessment includes two established questionnaires focused on organizational capacity to implement EBPs. The Implementation Leadership Scale (ILS) assesses the degree of organizational support for EBPs, and the Implementation Climate Scale (ICS) assesses staff’s impressions on expectations and support for effective EBP implementation through their policies, procedure, and behaviors [93].

### Life goals fidelity

Because this study is designed to assess real-world implementation, minimally invasive measures to assess LG fidelity will be based on provider logs sent via a secure web-based form to study staff on a weekly basis. Study staff members will also collect confirmatory data from patient surveys on receipt of LG sessions at the patient level. The fidelity monitor calculates a total score based on number of sessions completed by each patient and the percentage completing five of six sessions. Average patient completion of sessions of ≥75% was associated with improved mental health-related quality of life [49].

### Secondary aims: facilitation implementation and costs

Data on the implementation of EF and EF/IF will be collected by study staff members using previously established assessments for monitoring implementation activities [40],[43],[94]. Study staff members will interview LG providers, EF, IF, and site clinic leaders regarding barriers and facilitators to LG, REP, and EF/IF strategies, interactions between the EF and IFs, and specific strategies EFs and IFs use to facilitate LG uptake across different sites.

Implementation strategy costs will be ascertained by the study staff members based on study staff logs of REP activities, and LG provider and EF and IF logs based on a standardized list of activity categories (Table 3). EF and IF time is likely to account for the vast majority of costs associated with the implementation strategies, including site employees (LG provider time on training and LG implementation, IF time at meetings) as well as project staff (REP package development, training/program assistance, EF Facilitator time in site follow-ups). All costs will be multiplied by personnel wage rates including fringe. Patient-level costs will also be estimated from self-reported utilization survey data on inpatient, ER, and outpatient use. Costs will be assigned using Current Procedural Terminology (CPT) codes, and a relative value unit (RVU) weight will allow us to use the Medicare Fee Schedule to calculate a standardized cost in US dollars for each service, adjusted for annual levels of inflation using the consumer price index.

**Table 3:** **Summary of specific implementation strategy activities**

Implementation component	Tasks
REP	Pre-implementation meetings with study staff and consultants to finalize implementation design
	Study staff finalize plans for data security and related research logistics
	Creation of LG website for use by providers (software development, programming)
	Creation of packaged, user-friendly manual and customized training program
	Conduct organizational needs assessment at sites
	Pre-implementation in-service meetings with site leaders
	Identify LG providers at each site
	Identify potential Internal Facilitators (IF) at each site
	Regional 8-h LG trainings
	As-needed program support assistance via phone or email
	Monitor LG uptake via secure web-based reporting
	Disseminate monthly implementation progress reports to all sites
	Sites that are non-responsive are randomized to receive External Facilitation (EF) alone or EF + IF in combination
	Send out quarterly program updates that include bright spots and success stories for overcoming barriers in implementation.
External Facilitation (EF)	Weekly phone calls with facilitators and technical assistance staff to transfer knowledge and strengthen partnership
	EF contacts LG provider/s at non-responsive sites at least monthly for 6 months to develop rapport and understanding of site
	Assist LG providers in identifying what specific actions they can take to implement program
	Works with LG provider to set measurable goals for site implementation and monitors progress to support uptake
	Provides specific guidance on overcoming barriers in implementing LG components by aligning LG’s strengths with LG’s available influence at the site
	Facilitators refer LG providers to existing resources including ongoing LG program assistance when necessary
	Weekly meetings or calls between EF and TA to ensure open communication and collaboration
	Facilitator weekly consultation meeting with facilitation experts
Internal Facilitation (IF)	EF contacts IF at site to provide overview of internal facilitation role and documentation requirements for 6 month period
	IF, EF, and LG hold biweekly calls to develop rapport, review program progress and identify barriers and facilitators to LG uptake
	IF works with LG provider to develop measurable goals to LG uptake.
	IF identifies local site priorities per leadership that align with LG program.
	IF helps LG provider identify other LG program champions.
	EF helps IF and LG provider summarize and describe added value of LG to leadership and site providers (e.g., consistency with other initiatives, support from leadership)
	IF refers LG provider to LG program assistance as needed and appropriate
	IF, EF, and LG provider summarize progress and present to leadership
	IF works with EF and LG provider to develop business and sustainability plans and presents to leadership

### Analyses

Intent to treat analyses will be performed. The primary analysis will compare interventions in non-responding sites beginning with REP + EF/IF versus interventions beginning with REP + EF on longitudinal patient-level change in number of LG sessions received, SF-12 mental health-related quality of life scores, and PHQ-9 scores. This analysis is a two-sample comparison of cells A + B vs. C + D + E (Figure 2). For this analysis, the longitudinal outcomes will be measured at months 6 (pre-randomization), 12, 18, and 24. The primary contrast is the between groups difference in change from month 6 to month 18. The follow-up contrast at month 24 will also be examined in this and all subsequent analyses.

The primary aim analysis for health-related quality of life (the primary longitudinal outcome) will use linear mixed models (LMM; [95]), also known as random effects models. The unit of analysis is the individual patient within a site (recall that approximately 20 individuals will be identified prior to randomization). LMMs use all available measurements, allowing individuals to have an unequal number of longitudinal observations and producing unbiased parameter estimates as long as unobserved values are missing at random. The analysis will fit a three-level (repeated measures for each individual clustered within site) LMM with fixed effects for the intercept, time, group, and a group-by-time interaction term, where group is an indicator of REP + EF/IF vs. REP + EF. The LMM will include random effects for site and time, an unstructured within-person correlation structure for the residual errors, and it will adjust for state and type of practice (primary care or mental health site). LMMs similar to the above will be conducted for the secondary patient-level outcomes: change in number of LG sessions received, functional impairment, and mood symptoms.

Secondary aim analyses will be conducted to determine whether continuing REP + EF vs. augmenting with REP + EF/IF leads to changes in outcomes, among sites who are non-responsive to REP + EF at month 12. This secondary analysis is a comparison of cells B vs. C (Figure 2). Outcomes will be examined using an LMM similar to that described above, except (a) including only the subset of sites that do not respond at month 12 to REP + EF, (b) using monthly longitudinal outcomes from month 12 to month 24, and (c) the LMM will use dummy indicators for time (i.e., time-saturated model since there are only three measurement times for each longitudinal outcome). The longitudinal course of discontinuing REP + EF (or REP + EF/IF) will also be examined at month 12 among sites that are responsive at month 12.

Other secondary aim analyses include comparison of EF and EF + IF costs over time using a standardized list of REP and EF/IF activities (Table 3), as well as assessment of organizational change over time [96]. Additional exploratory analyses will also compare the different adaptive interventions embedded in the SMART design (Figure 2) in terms of changes in longitudinal outcomes using methods based on Robins and colleagues [97],[98].

Missing values may occur in outcomes due to dropout or inability to reach patients for follow-up (anticipated 10% attrition). A thorough investigation of mechanisms for missing data will be carried out and will be dealt with using multiple imputation procedures [99]. In stability analyses, data will be analyzed with and without the multiple imputation strategy. Any discrepancies will be reported and carefully examined.

### Sample size and power

The estimated sample size for this study is based on a comparison of between groups (REP + EF/IF versus REP + EF) in changes in the most conservative effect size estimate (mental health-related quality of life scores) between month 6 and month 18 (Cohen’s *D* = .23). This is a two-sample comparison of patients within sites in cells A + B + C versus D + E (Figure 2). To account for the between-site variation induced by the within-site correlation in quality of life outcomes, we inflate the variance term in the standard sample size formula by 1 + (*n* - 1) × ICC, where ICC is the site inter-class correlation coefficient for the SF-12 mental health-related quality of life component score. Based on our previous randomized controlled implementation trial [40],[43], the site ICC was estimated at .01. Using a two-sided, two-sample *t* test, a Type I error rate of 5%, and with 60 sites (30 sites randomized to REP + EF/IF versus 30 sites randomized to REP + EF) and 16 patients per site after accounting for attrition, we will have 94% power to detect clinically significant changes in quality of life scores, assuming an ICC = .01. Based on previous data, with SD = 8.35 for the MH-QOL component score, this effect size corresponds to being able to detect a clinically meaningful difference of at least 4 units in MH-QOL score.

### Trial status

Sites will be identified and participation confirmed by October 2014. Site training and REP procedures will begin in the fall of 2014. A timeline of implementation activities is provided in Table 3.

## Discussion

To date, this will be one of the largest randomized controlled trials to develop a site-level adaptive implementation strategy. This is also one of the first studies to test the augmentation of an established implementation strategy (REP) using REP + EF/IF or REP + EF alone among sites that exhibit non-response after 6 months. This study will determine whether augmentation of the implementation strategy is needed (e.g., REP + EF/IF) or whether in some circumstances withholding augmentation may result in a delayed implementation effect of REP + EF alone among non-responsive sites. Second, this study will utilize a novel SMART design developed by the study investigators [51] to accomplish this implementation trial. SMARTs allow efficient comparison of the overall impact of receiving different intervention augmentation strategies over time and incremental costs of one intervention strategy over another in improved outcomes among non-responsive sites. In addition, this design allows the potential to determine the added value of implementation strategies applied to real-world treatment settings.

There is a paucity of research on effective implementation strategy that improves EBP uptake and ultimately patient outcomes, notably in smaller, community-based, safety net practices, which serve a substantial proportion of patients with mood disorders. There have been few rigorous trials of implementation strategy to promote the uptake of EBPs in community-based practices [1],[35]. Previous implementation studies have focused on highly organized practices such as the VA or staff-model HMOs and mainly involved intensive implementation strategy that might not be feasible to apply to smaller, lower-resourced practices. Among the frameworks that guide implementation efforts (e.g., [100]), few have been operationalized sufficiently to enable community-based practices to enhance EBP uptake.

Adaptive Implementation of Effective Programs Trial (ADEPT) represents a growing cadre of rigorous yet real-world trials that involve site-level randomization of quality improvement strategies to augment the implementation of evidence-based practices. Specifically, informed consent from participants is not required per the recommendation of the IRB because the focus of ADEPT is on the use of implementation strategies at the site level, above and beyond available resources to disseminate effective programs, in order to assist existing providers in adopting an evidence-based practice. Moreover, the delivery of the evidence-based practice to patients is not altered or controlled at the sites by the study team, and patient clinical assessment includes measures that are used across many of the practice settings as part of routine clinical care. These types of studies have high generalizability to real-world settings because they reflect the pragmatic strategies needed to improve healthcare delivery and foster learning health systems [101],[102]. ADEPT evaluates the use of an evidence-based intervention (i.e., life goals) that is superior to usual care from the patient perspective while testing an implementation strategy (facilitation) that is beneficial to providers and practices by improving their ability to adopt such evidence-based practices [102]. Designation as a QI trial does not remove IRB regulatory oversight but instead reduces the burden on researchers (costs) and patients (time) to participate in written informed consent and formal survey assessment meetings which do not reflect real-world care but instead introduce potential patient selection biases that undermine patient participation in the intervention [101],[102].

REP uses key tactical strategies that can promote effective EBP adoption in community-based providers. However, these providers may face multiple organizational barriers at their sites including competing demands or lack of experience in garnering leadership buy-in that are beyond the scope of toolkit dissemination, structured training, or initial performance feedback [10],[15],[16],[103]. Addressing these barriers may require strategic thinking and multilevel organizational alliances [104],[105] that engage providers in supporting and owning the implementation [106]-[108]. While leadership support in EBP adoption is important [109]-[111], involvement and buy-in from frontline providers are also crucial to EBP sustainability [94],[109],[112]-[118]. Hence, REP may need to be augmented to address these organizational barriers to adoption [119],[120] and to ultimately show value of the EBP through the triple aim (improving patient outcomes, experience, lowering costs) [121].

Our study will determine the added value of augmenting implementation programs through Facilitation on EBP uptake, sustainability, and patient outcomes, ultimately determining the public health impact of implementation strategies for persons with mental disorders. Akin to stepped treatment for patients, adaptive SMART designs provide a tailored and potentially cost-effective approach to augmenting standard implementation programs based on the specific needs of a site. This approach is needed to better understand how to improve EBP uptake and patient mental health outcomes using Facilitation, whereby REP is augmented given early signs of site non-response. While REP might be sufficient for some sites in adopting EBPs, our preliminary studies and prior research [122],[123] suggest that the majority will need additional assistance. Moreover, sites initially not responding to REP (i.e., limited adoption of EBPs) were unlikely to do so in the future. IF is more expensive for sites to implement, as the additional customization and relationship building across individual sites due to variations in culture, climate, and capacity can be time consuming [113]. It is also unclear how long Facilitation is needed to achieve its effect [103],[109],[117]. Adaptive interventions involving REP and augmentation through Facilitation are needed to determine the added value of REP + EF/IF on improved patient outcomes, how long Facilitation need to be continued to achieve outcomes, and the cost-effectiveness of REP + EF/IF.

While this study has a number of strengths, including the use of a novel implementation study design within community-based practices, there are key limitations that need to be considered. First, there is the potential for self-selection of site early adopters which can limit generalizability. The involvement of sites most willing to participate in studies is inevitable with any implementation design. We have mitigated this potential selection effect by inviting all sites from networks of practices through state associations from across the country. In addition, LG uptake and fidelity measures do not include direct observations of sessions. In order to conduct an implementation trial that reflected real-world considerations, we chose to use indirect methods to measure LG uptake, and cost considerations precluded us from conducting in-person observations. Nonetheless, because the study was considered non-regulated (non-research), there is potential for increased generalizability especially if patient dropout is minimized because they will be assessed as part of routine clinical care. In addition, there is a chance for contamination between the EF and IF implementation strategies, especially if a less engaged IF might promote the EF to become more active in assisting the LG provider in identifying barriers and facilitators to program uptake. Finally, not all sites from each state participated, and those what are enrolled may not be representative of community-based practices nationwide.

## Conclusions

The results of this study will inform the implementation of evidence-based practices across sites in two states, as well as how to conduct practical implementation studies in real-world healthcare settings. This study also sets the stage for determining the added value of more intensive implementation strategies within sites that need additional support to promote the uptake of EBPs. Ultimately, adaptive implementation strategies may produce more relevant, rapid, and generalizable results by more quickly validating or rejecting new implementation strategies, thus enhancing the efficiency and sustainability of implementation research and potentially lead to the rollout of more cost-efficient implementation strategies.

## Authors’ contributions

AMK provided funding for the study, developed the implementation methods, and drafted the manuscript. DA, DE, MSB, and JCF provided input on the study design and feedback on paper drafts. DEG, MRT, JEK, LIS, and JW provided literature and relevant background on the implementation model, as well as critically reviewed subsequent drafts. MSB, LIS, DE, and JK provided input on the adaptation of the clinical program and outcomes assessment design. KMN, JEK, JK, SAM, and JW operationalized the adaptive interventions and reviewed paper drafts. KMN, DM, JF, SM, and JK reviewed the study drafts and provided components of the clinical intervention. All authors read and approved the final manuscript.

## Authors’ original submitted files for images

Below are the links to the authors’ original submitted files for images. Authors’ original file for figure 1 Authors’ original file for figure 2
