# Supplementary figures and images for: Protocol: Adaptive Implementation of Effective Programs Trial (ADEPT): cluster randomized SMART trial comparing a standard versus enhanced implementation strategy to improve outcomes of a mood disorders program
Source: Implement Sci. 2014 Sep 30;9:132. doi: 10.1186/s13012-014-0132-x (PMC4189548; doi:10.1186/s13012-014-0132-x)

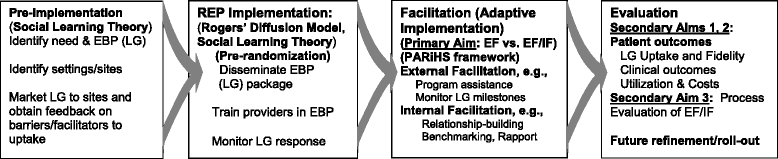

Supplement: Supplementary file 1 — Authors’ original file for figure 1 [file 13012_2014_132_MOESM1_ESM.gif]

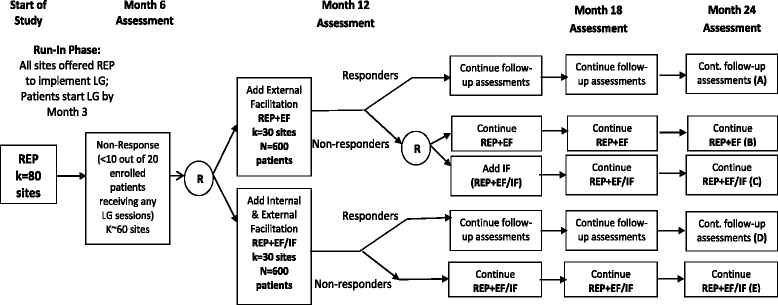

Supplement: Supplementary file 2 — Authors’ original file for figure 2 [file 13012_2014_132_MOESM2_ESM.gif]
